# Hand-Held Zoom Micro-Imaging System Based on Microfluidic Chip for Point-of-Care Testing (POCT) of Vaginal Inflammation

**DOI:** 10.1109/JTEHM.2021.3054556

**Published:** 2021-01-26

**Authors:** Ning Yang, Jingxin Peng, Liang Wu, Xue Han, Naila Shaheen, Xiaobo Zou

**Affiliations:** 1 Department of Electrical and Information EngineeringJiangsu University 12676 Zhenjiang212013China; 2 Department of MedicineJiangsu University 12676 Zhenjiang212013China; 3 Department of Food and Biological EngineeringJiangsu University 12676 Zhenjiang212013China

**Keywords:** Bacteria detection, inflammation assessment, micro-imaging system, microfluidic chip, POCT

## Abstract

Background: Vaginitis is a common and very private disease, and the current diagnosis is a frequent go to the hospital for testing. Objective: In order to improve the convenience and speed of detection, in this paper, we have developed a hand-held zoom micro-imaging system based on a microfluidic chip for point-of-care testing (POCT) of vaginal inflammation. Methods: This system consists of a microfluidic chip, an optical system and a hand-held zoom system assembled with a mobile phone. In terms of hardware, we designed a self-priming microfluidic chip, which can realize automatic sampling and full mixing of samples. We have also developed an optical system that can be adapted to smartphones, which has a lens group with a 37x magnification function and equipped with a zoom system with a focus range of 4mm to 6mm. In terms of software, we proposed an APP that can accurately identify cocci and can determine the inflammation level. Results: Compared with the recognition rate of the observers in the hospital, the detection accuracy of the portable recognition system is 95%, and after testing the clinical samples, the results were completely consistent with the hospital diagnosis results. The detection limit was 500 CFU / ml, which the relative error was (0.9 ± 0.3) %, and recognition time is 7 seconds. Conclusion: This system is definitely suitable for women’s point-of-care testing (POCT).

## I. Introduction

Vagina is an essential micro-ecological region in women’s body [1]. It is an integral part of the female reproductive tract and the only channel connecting with the outside world, and its stability is closely related to women’s health [2]. According to statistics, approximately 10 million physician office visits each year are attributed to vaginal discharge in the United States [3]. However, bacterial vaginitis is a very private disease and has a very high recurrence rate [4], Chinese women are often unwilling or have no time to go to the hospital for examinations due to traditional ideas, which may cause serious consequences [5]. Therefore, it is crucial to develop a system that allows women to complete vaginal inflammation tests at home.

For the detection of bacterial vaginosis, the traditional detection methods are smear microscopy, amine test, culture and biochemical method. J. D. Davis *et al.* used Gram stain to detect bacterial vaginitis. They proposed that it is more sensitive to bacterial vaginosis than conventional smear microscopy [6]. However, vaginal secretions are not only a series of bacteria but also a large number of epithelial cells. Smear microscopy has observation errors, requires a professional operation and is cumbersome. D. S. Jack *et al.* measured biogenic amines by ion mobility spectrometry to diagnose vaginal infections [7]. But they need to use gas chromatography (GC) or high-performance liquid chromatography (HPLC), which are often expensive, complicated to operate, and not suitable for rapid clinical testing. P. T. Jason *et al.* used real-time PCR and pyrosequencing to detect and identify Candida species associated with Candida vaginitis [8], this method has high precision, but it also has certain defects, such as long detection time, which can only be used in the laboratory. In general, the detection of vaginal secretions relies on complex, high-cost specialized instruments or cumbersome biochemical detection methods, which are mostly used in hospitals or laboratories, limiting the application in home testing or rapid clinical testing.

Compared with the traditional complex detection, smartphone-based detection is currently a more popular disease diagnosis method [9]. A. Orth *et al.* used a camera flash and ambient light to design a dual-mode mobile phone microscope to detect microorganisms [10]. Still, they did not develop the focusing mode, and the resolution was meager, so it was not suitable for the detection of small microorganisms. G. Chhavi *et al.* designed a mobile phone-based ultraviolet fluorescence microscope for identifying fungal pathogens [11]. However, their microscope cannot adjust the focal length, so it cannot achieve accurate identification at different levels, and the detection of microorganisms is incomplete. W. M. Kadlec *et al.* developed a cellphone-based microphotometric system for rapid antimicrobial susceptibility testing [12]. However, they are not equipped with microfluidic chips, the operation steps are complicated and a large of reagents are required, which is not suitable for on-site detection. For microfluidic chip technology, many researchers have conducted in-depth discussions on chip design. Whether it is a paper chip [13] or a PDMS chip, after designing different functional structures [14], the automatic processing of samples can be realized, which reduces many experimental steps and is very portable [15]. Therefore, microfluidic chip plays an important role in medical diagnosis and other fields.

With the rapid development of microfluidic technology and 5G network, smartphones detection become the hot spot and key to point-of-care testing (POCT) [16], which can provide diagnostic data in a more timely and effective manner, as well as detect without the need for trained personnel and advanced infrastructure, which may make it an ideal candidate for overcoming the diagnostic difficulties caused by lack of resources [17]. L. Fengyun *et al.* used plastic micro-chips with micro-pit array ($\mu $PACs) and a mobile phone for point-of-care testing (POCT) of HIV capsid p24 antigen [18]. I. Shazia *et al.* proposed a smartphone-based sickle cell disease detection and monitoring methods for point-of-care testing (POCT) [19]. T. Junming *et al.* proposed a point-of-care testing (POCT) method based on quantum dots for measuring procalcitonin in finger pricking and venous whole blood samples [20]. However, they have not developed a suitable APP in the mobile phone, even if the specificity and sensitivity of their methods are excellent.

Here, we designed a self-priming microfluidic chip, which can automatically inject samples, save sampling time, and mix samples adequately. Besides, we invented a hand-held mini-microscope device, which has an optical magnification system adapted to a mobile phone and a focusing system with a range of 2 mm. It is used with this device to obtain microscopic images of vaginal secretion samples. At the same time, a rapid identification APP for vaginal secretion bacteria was proposed. The system designed in this article is only the size of a palm, with high-definition resolution and portability, it can be used for rapid detection of vaginal secretions in point-of-care testing (POCT).

## II. Materials and Methods

### A. Sample Preparation

To simulate the inflammation level of vaginal secretions in women, we prepared Staphylococcus aureus (S. aureus) and Escherichia coli (E. coli). The two centrifuged bacteria were diluted in PBS solution to the same concentration. According to the four levels of inflammation, the ratio of S. aureus and E. coli was 1:9, 4:6, 6:4, 9:1, respectively. At the same time, we also obtained four clinical samples from Jiangbin hospital (Zhenjiang, Jiangsu Province, China), two of which were abnormal.

### B. Design of Self-Priming Microfluidic Chip

The proposed self-priming microfluidic chip was fabricated using standard soft lithography [21]. The chip can achieve sample self-priming and sufficient mixing function, which is made of glass and PDMS.

For the self-priming of the sample, a hydrophilic material [22] (Mesophilic-2000, PEG) was injected into the microchannel. After staying for one minute, the liquid in the microchannel was slowly discharged with air using a pipette gun (100-1000ul). At this time, hydrophilic nano coating is formed inside the microchannel.

**FIGURE 1.**
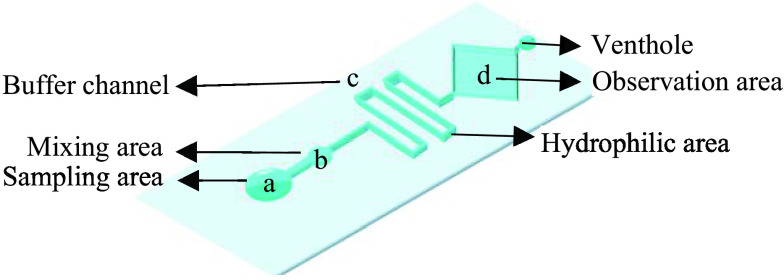
Microfluidic chip structure design.

The structure of the chip is designed to better mix samples, and its size is $2\times 5$ cm. As shown in Figure 1, a is the sampling area (the diameter is 3 mm), b is the mixing area used for sample diffusion (the diameter is 2 mm), c is a buffer channel for uniformly mixing microorganisms (the width is 2 mm), and d is the observation area (the diameter is 2 mm).

**FIGURE 2.**
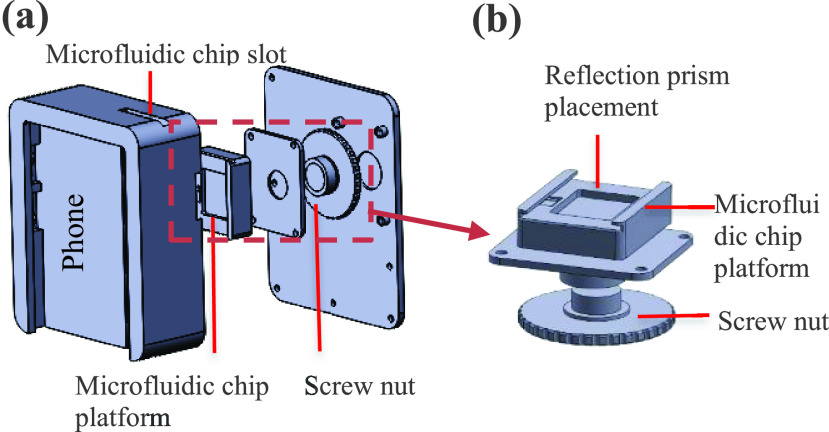
Installation drawings of solid works.

### C. Optical System Design

**FIGURE 3.**
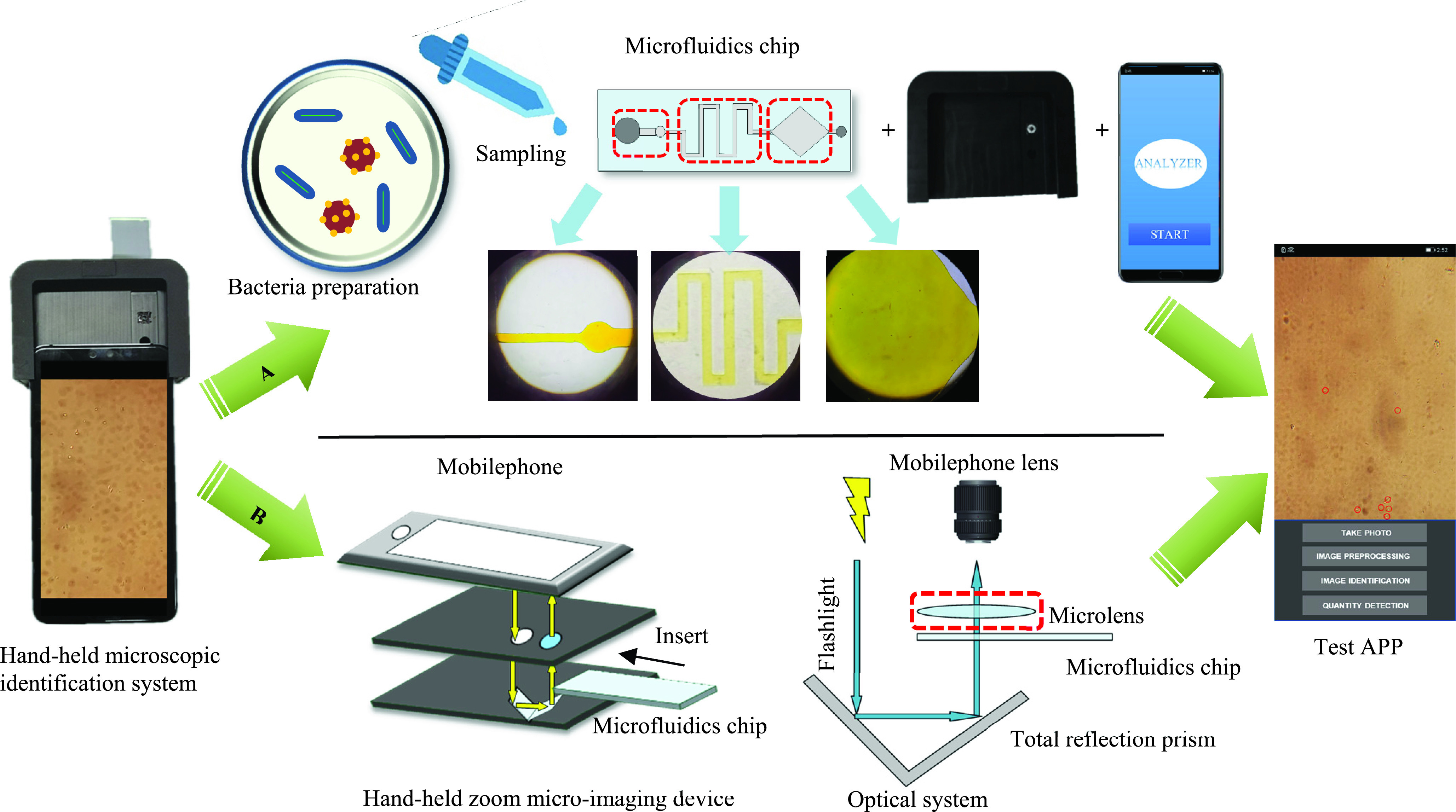
Hand-held microscopic identification system schematic. (A) Schematic diagram of system operation flow. (B) Schematic diagram of the internal principle of the hand-held microscope.

The optical system is shown in Figure 3. It consists of a micro-lens group, a smart phone flash, and a total reflection prism. In order to realize the function of optical amplification, we use the flash of the smartphone as the light source. After the light source is vertically irradiated on the total reflection prism, the optical path changes, and the reflected light path passes through the sample slide and the micro-lens group, finally, an enlarged sample micrograph is obtained, which is captured by a smartphone. The microlens group is composed of multiple high-power microlenses with 37 times magnification.

### D. Hand-Held Zoom Micro-Imaging Device Design

The hand-held zoom micro-imaging device is the core part of the whole system, and it is customized according to the smart phone (P20, Huawei, China). It has an optical system inside, including a movable microfluidic chip platform and total reflection prism. The platform and the prism are fixed together and connected to the nut. The mobile phone can be directly connected to the device. By turning the external nut, the microfluidic chip platform can be moved up and down inside. Since the focal length of the lens is constant, but the focus is not in the ideal position, it is necessary to change the position of the microfluidic platform to find the clearest imaging point, as shown in Figure 2. Each part is drawn using solid works software, then 3D printed, and finally, each part is installed together.

### E. Construction of a Hand-Held Microscopic Identification System

The schematic diagram of the hand-held microscopic identification system is shown in Figure 3. It includes hand-held micro-imaging device, microfluidic chips, and smartphones. When the system is working, the sample is sampled first, and the configured sample solution is dropped on the self-priming microfluidic chip, after waiting for the microfluidic chip to absorb the droplets evenly, insert the chip into the slot of the hand-held micro-imaging device. Then assemble the camera and the device, open the camera of the phone, and turn on the flash to capture the microscopic image of the sample slide. Finally, the mobile phone APP detects and recognizes.

### F. Algorithm Research of Hand-Held Microscopic Identification System

The vaginal inflammation can be divided into four grades, and the criteria for determining the level of inflammation are as follows [23]: I degree is mainly vaginal bacillus, and a large number of epithelial cells are visible; II degree has some vaginal bacilli, epithelial cells are also visible, and some coccus and bacteria are also present; III degree only a small amount of epithelial cells, but a large number of coccus and other bacteria; IV degree no epithelial cells, almost all coccus and a large number of other bacteria. I to II degrees are normal, and III to IV degrees are morbigenous. Therefore, the number of cocci and bacilli is the key to distinguishing the inflammation. The level of inflammation is judged, as shown in table 1.

**TABLE 1:** Inflammation Level Judgment Standard

Level	Vaginal bacillus	Cocci	Bacillus	Epithelial Cells	Pus or white blood cell
I	++++	–	++++	++++	0~5/HP
II	++	–	++	++	5~15/HP
III	–	++	–	–	15~30/HP
IV	–	++++	–	–	>30/HP

After the sample photos are acquired by the mobile phone, the algorithm performs image preprocessing, including filtering, binarization and boundary detection. The key to judging the inflammation is the ratio of cocci and bacillus. We used an image recognition algorithm based on chain code technology to recognize the contours of cocci and bacilli. The outlines of the two extracted bacteria are roughly round and rod-shaped, and the cocci can be determined by calculating the roundness of each outline [24]. The formula is as follows:

\begin{equation*} C=\frac {L^{2}}{4\pi A}\tag{1}\end{equation*}

L is the target perimeter, A is the target area, and C is how close the target is to the circle. When $C=1$, it is a circle [19].

For area A, which is also the area surrounded by the chain code, there is the formula:

\begin{equation*} A=\sum \nolimits _{i=1}^{n} {a_{i0}\left({y_{i-1}+\frac {1}{2}a_{i2}}\right)}\tag{2}\end{equation*}

In the formula, $y_{i}$ ($i=0, 1, 2,\ldots, n$) is coordinate, and $y_{0}$ is the starting coordinate, $a_{i0}$ and $a_{i2}$ are the components of the length of the i-th ring of the chain code at $k=0$ (horizontal) $k=2$ (vertical).

For a certain round chain code: $x=x_{0}$, $y=y_{0}$, $c_{i}=c_{1}$, $c_{2}$, $c_{3},\ldots, c_{\mathrm {n}}$. the following iteration formula is obtained according to the calculation formula (2):

\begin{align*} A=&A+dx\left ({c_{i} }\right)\times \left({y_{i-1}+\frac {1}{2}dy(c_{i})}\right) \tag{3}\\ y_{i}=&y_{i-1}+dy(c_{i})\tag{4}\end{align*}

Through continuous iterative calculations, find the bacteria with a circular outline. Since the size of the cocci is basically the same, and there is an obvious circular outline and a high-brightness center, the area and gray value of the circular outline can be calculated to identify and distinguish the cocci. We transplant the algorithm into the Android program, and the mobile phone can complete image processing and recognition.

## III. Results and Discussion

### A. Self-Priming Microfluidic Chip

**FIGURE 4.**
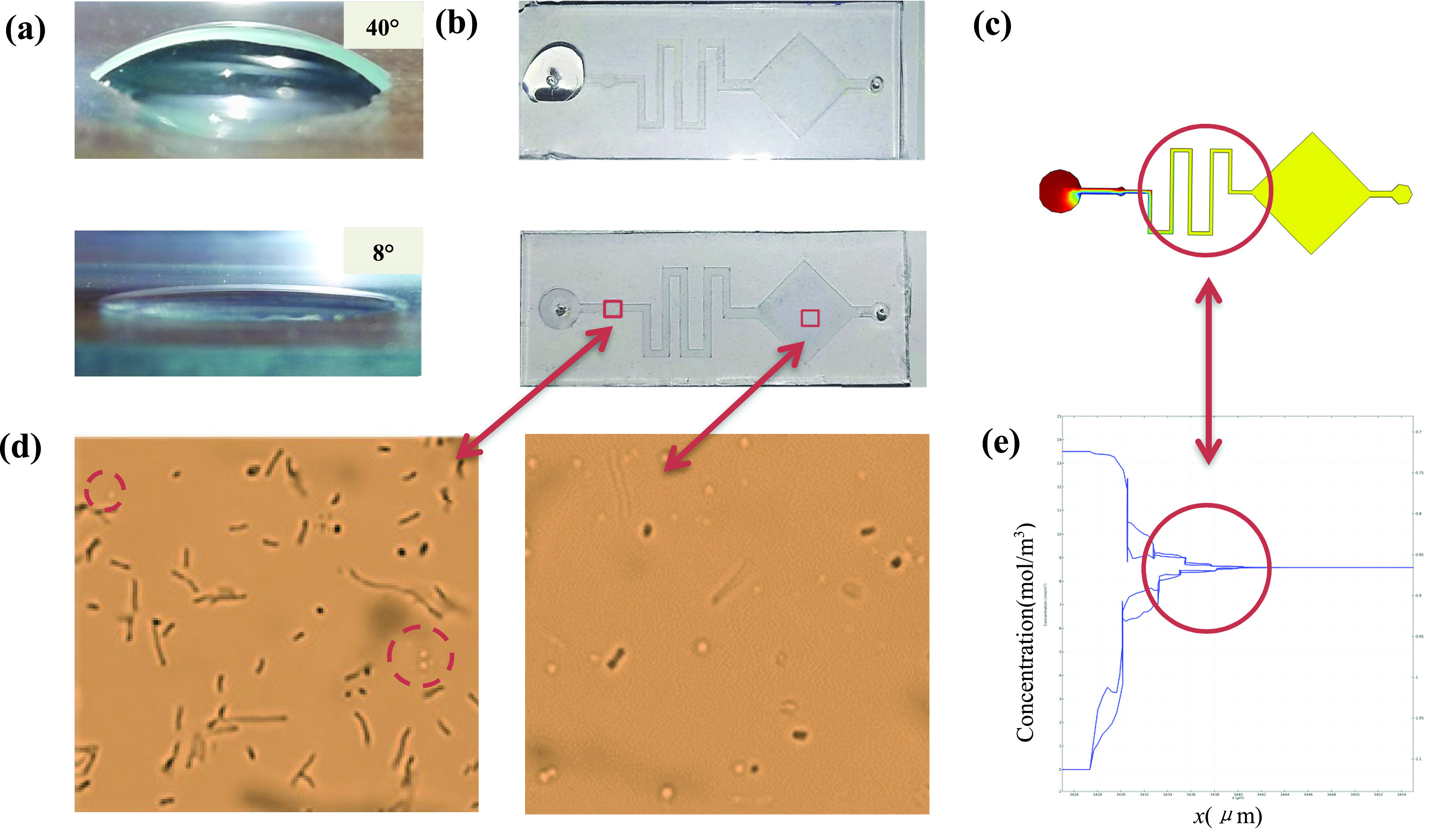
Self-absorption and mixing effect of microfluidic chip. (a) Comparison of hydrophilic angle. (b) Self-priming effect comparison. (c) Mixed effects simulation. (d) Results of microfluidic chip mixing. (e) Variation law of mixed concentration.

Due to the action of the hydrophilic material, the contact angle of the water droplet chip surface changes. Through measurement, the contact angle of the chip without treatment is about 40°, and the angle after treatment is about 8°, as shown in Figure 4(a).

The structure of the designed microfluidic chip was simulated by COMSOL to simulate the fluid concentration in the channel, in order to verify its mixing effect and sample uniformity. I t can be seen from Figure 4(c) and (e) that the inlet of the microchannel has two different concentrations of fluid. After passing through the buffer zone, the two concentrations of fluids are completely mixed, the concentration difference is reduced and eventually becomes zero.

In order to test the self-aspiration and mixing effect of the chip, we use two pipettes (0.5-$10~\mu \text{l}$, S. aureus and E. coli) to simultaneously add two samples with equal amounts and equal concentrations to the injection port. As shown in Figure 4(b), the untreated chip retains liquid at the entrance and difficult to flow into the chip without external force, while the processed chip can quickly and automatically suck liquid. (The video of chip self-priming has been uploaded). Figure 4(d) reflects that when there is no mixing, E. coli is obviously more than S. aureus, but after passing through the mixing area, they are evenly distributed.

### B. System’s Spatial Resolution and Field of View (FOV)

**FIGURE 5.**
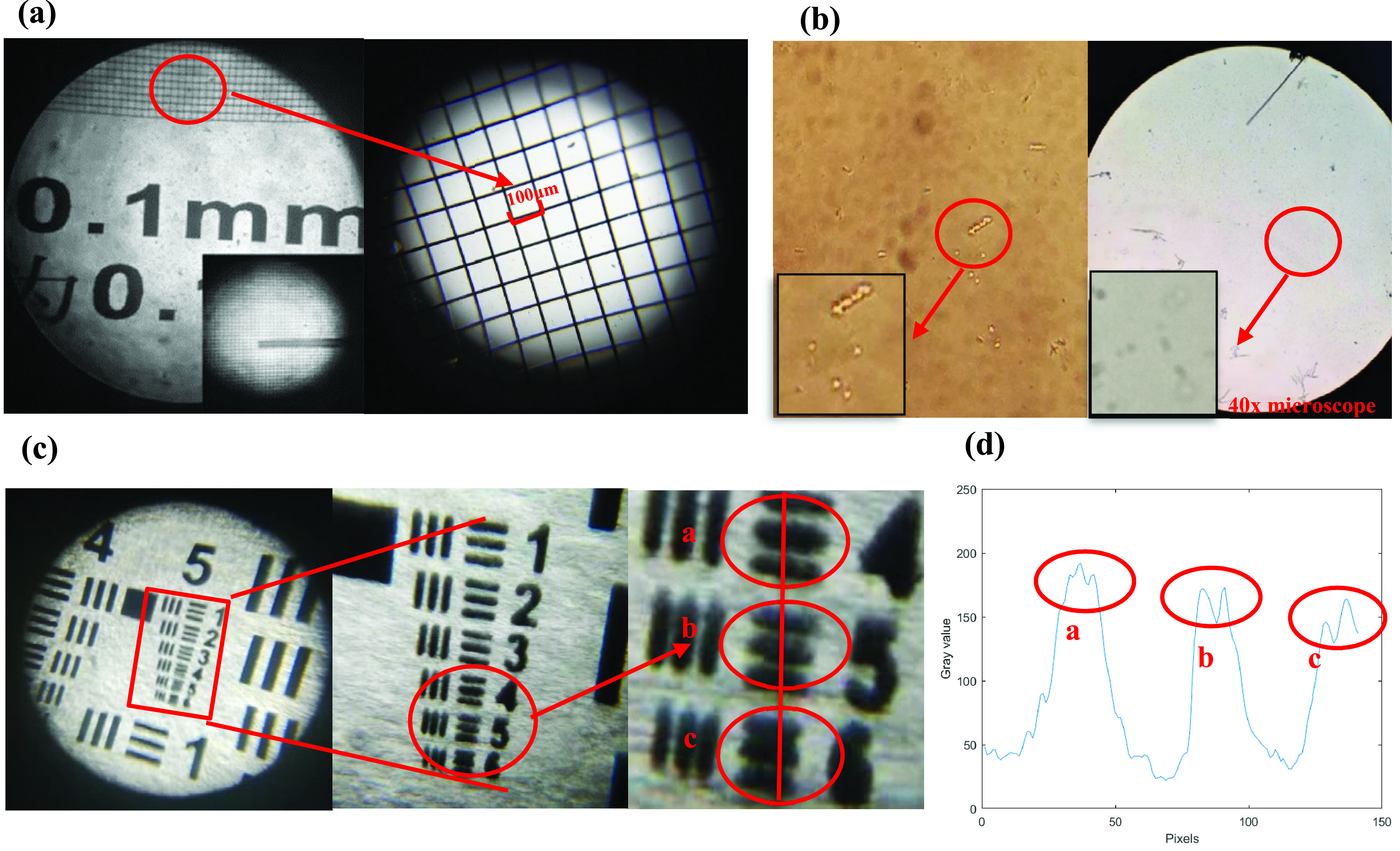
Field of view and resolution map of optical system. (a) Image of the stage micrometre that was used to determine the FOV. (b) Comparison of images under the handheld zoom micro-imaging system with images under a conventional microscope. (c) Image of the 1951 USAF resolution test chart taken with the hand-held zoom micro-imaging system. (d) The graph provides a cross section of a circle highlighting the lowest resolution group.

The optical system’s optical magnification is about 37 times, which can be combined with a smartphone to achieve digital amplification. It can be clearly seen under the resolution test card that the length of each grid is $100~\mu \text{m}$, as shown in Figure 5(a), and the entire field of view is much smaller than that of a traditional microscope. Still, the resolution is higher than a traditional microscope. Therefore, we compared the contrast between the hand-held micro-imaging system designed in this paper and the conventional microscope. As shown in Figure 5(b), on the right is a sample image magnified by a Nikon inverted microscope (40x mirror), and on the left is a magnified image of the same sample by the hand-held micro-imaging system designed in this paper. It can be seen that the sample field under the traditional microscope is vast, but the bacteria are small and cannot be seen clearly. The system designed in this paper has a small field of view, but the morphology of cocci and bacilli can be clearly seen.

We determined the spatial resolution of the hand-held zoom micro-imaging system by the following methods: the hand-held zoom micro-imaging device was installed with the Huawei P20 mobile phone, and tested with the 1951 USAF resolution test chart. Imaging in a straight line, and then placing the resolution detection card under the lens, we moved the card until the line pairs on the chart became the focus, and finally took the image. We observed that our system was able to spatially resolve through flash lighting with 45.3 lp/mm (group 5, element 4), while 203.0 lp/mm (group 7, element 5). Therefore, this microscopic imaging system provides us with a comparable spatial resolution below $3~\mu \text{m}$, as shown in Figure 5(c) and (d).

We compared the actual field of view and the area of the field of view of the microscope with this equipment. The calculation formula of the actual field of view [25] is as follows:

\begin{equation*} F=\frac {N}{M}\tag{5}\end{equation*}

In the formula, $F$ represents the actual field of view, the unit is mm, which is the diameter of the field of view under the microscope, and $N$ is the field number, which is a given value, $M$ is the magnification of the microscope. A microscope with a 40x objective lens and a 10x eyepiece has a field of view of 22 and an actual field of view of 0.55mm (diameter d), so the field of view of the microscope is about 0.24mm^2^. However, the actual field of view of proposed system is about 0.059mm, and the field of view is about 0.0047mm^2^.

### C. Zoom System

Because the volume of microorganisms is too small, it is not guaranteed to be on the same level in the sample slide. The hand-held micro-imaging system designed in this paper can adjust the focus, and different focal lengths of focus can focus on varying levels of microorganisms. Take this person’s blood smear as an example, after calculating the sharpness of the images at these five different focal lengths, it can be seen that the image with the focal length of 1.37mm is the clearest, so the sharpness is the highest, as shown in appendix Figure 1. It needs to be emphasized that for different samples, the sharpest imaging focal length is different.

### D. Evaluation of Microscopic Characteristics

The result that can be obtained is that the field of view of the system is 51 times (under maximum magnification) smaller than a traditional microscope. Therefore, we converted the field of view of the system into the field of view of the microscope and compared the number of bacteria in two ways at different concentrations. Under the microscope, it was obtained by the plate counting method, and the mobile phone directly counted. The sample solution provided is a dilution of all S. aureus; the concentration range is 500–20000 CFU/ml. The concentration can be calculated by the total number of detected bacteria divided by the detected volume which is the multiplication of the sample chamber thickness and the total area of the detected FoVs [26]. It should be emphasized that the concentration calculated by this method is expressed as cells/ml, while the concentration of the sample is expressed as CFU/ml. These two indicate different counts when distinguishing cell viability, but they are the same for living cells. This article will not make a distinction.

**FIGURE 6.**
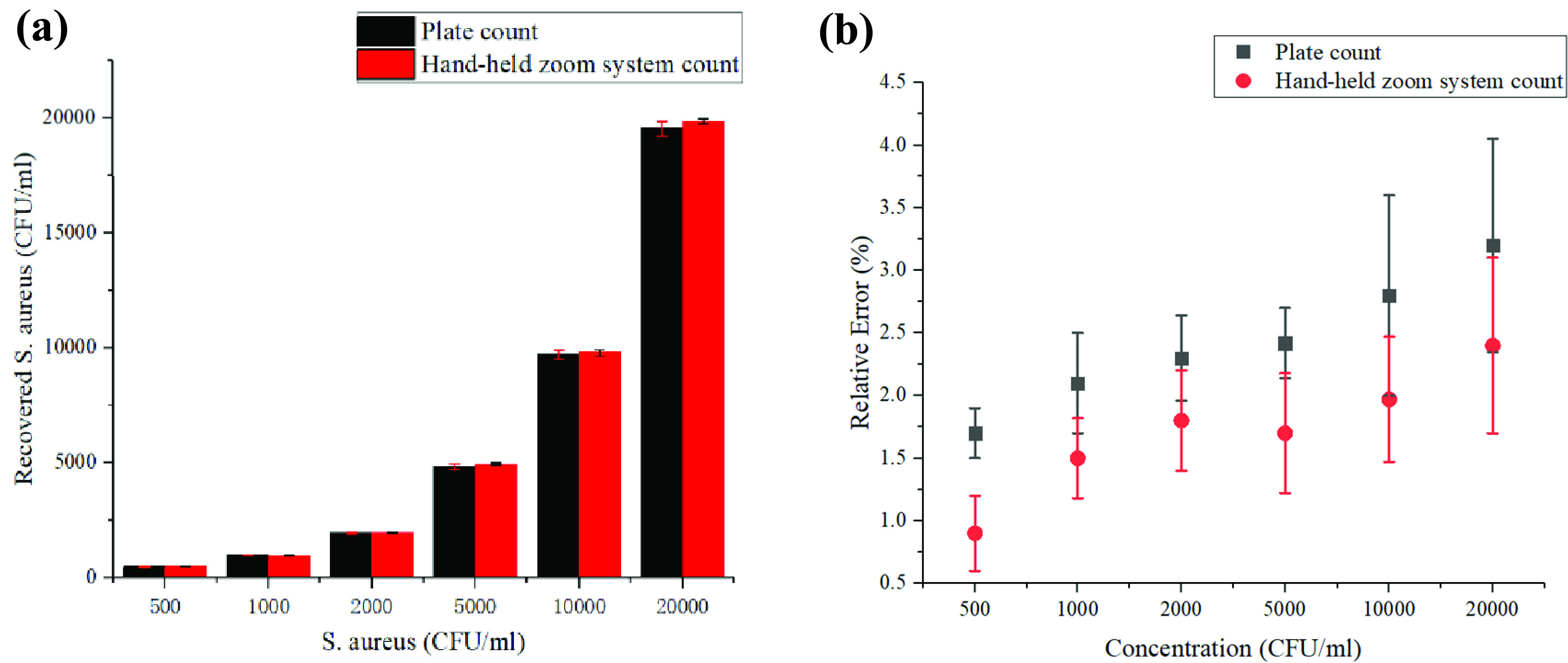
(a) Comparison of counting results between the plate counting method and the hand-held zoom system under different concentrations of S. aureus solution. (b) Concentration detection range.

Figure 6 shows that the calculated concentration results of the two methods are almost the same, and the linear regression rate is 0.9844. However, the data reflects that the larger the sample concentration, the greater the relative error, but in clinical diagnosis, the number of bacteria in female leucorrhea is difficult to reach a high concentration. Actually, a lower detection limit can meet the requirements of clinical testing. It can be seen from their relative errors that the proposed method has a lower detection limit, and the error is (0.9 ± 0.3) % in a suspension with a concentration of 500 CFU/ml.

### E. Identification of Cocci and Bacilli

**FIGURE 7.**
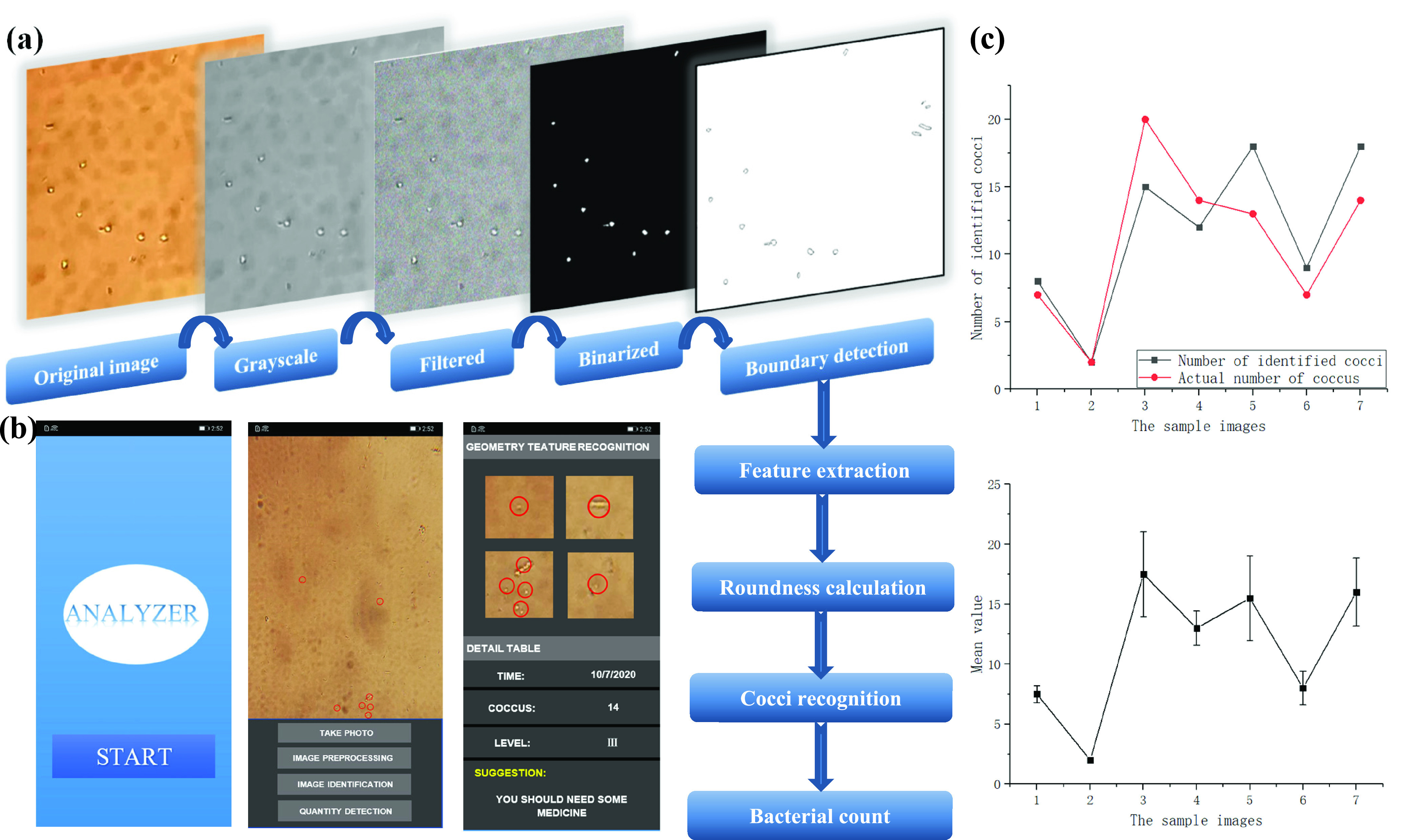
Bacterial identification, APP design and identification errors. (a) Image processing of bacteria. (b) APP interface and image processing flow. (c) Mobile phone identification error of cocci.

After the basic image processing of the sample pictures collected by the mobile phone, we perform feature extraction and roundness calculation on the extracted bacterial contours. Since the mobile phone algorithm can only calculate small-scale image data, the above steps are completed on the computer. The characteristic value of the bacterial contour is transmitted to the mobile phone algorithm, only the edge of the bacteria is calculated in the mobile phone. The designed APP interface is shown in Figure 7(b).

After analyzing 550 images of the simulated samples, it can be concluded that the accuracy of the Coccus identification algorithm is 90.76%, but the recognition error is 6.12%. However, inaccurate judgment in clinical medicine is also inevitable. As long as the number of identified cocci is greater than or equal to the number of actual cocci, the algorithm can also determine the level of vaginal secretions.

### F. Sensitivity of Vaginal Inflammation Level Diagnosis

In this experiment, the level of female vaginal secretions was studied. During the study, we simulated and equipped four levels of female vaginal secretions. Because the vaginal inflammation is mainly determined by the number of cocci, we only identified S. aureus at the same concentration ($1\times 10^{5}$ CFU/ml) in different proportions of the sample solution. A total of 100 samples were tested in this experiment.

Appendix Table 1 shows the test results of 10 randomly selected samples. It can be seen that the counting result of the recognition algorithm is linear with the counting result of the observer, and the result of the algorithm to judge the vaginal inflammation is the same as that recorded by the observer, the recognition accuracy was 95%, as shown in appendix Figure 2.

**FIGURE 8.**
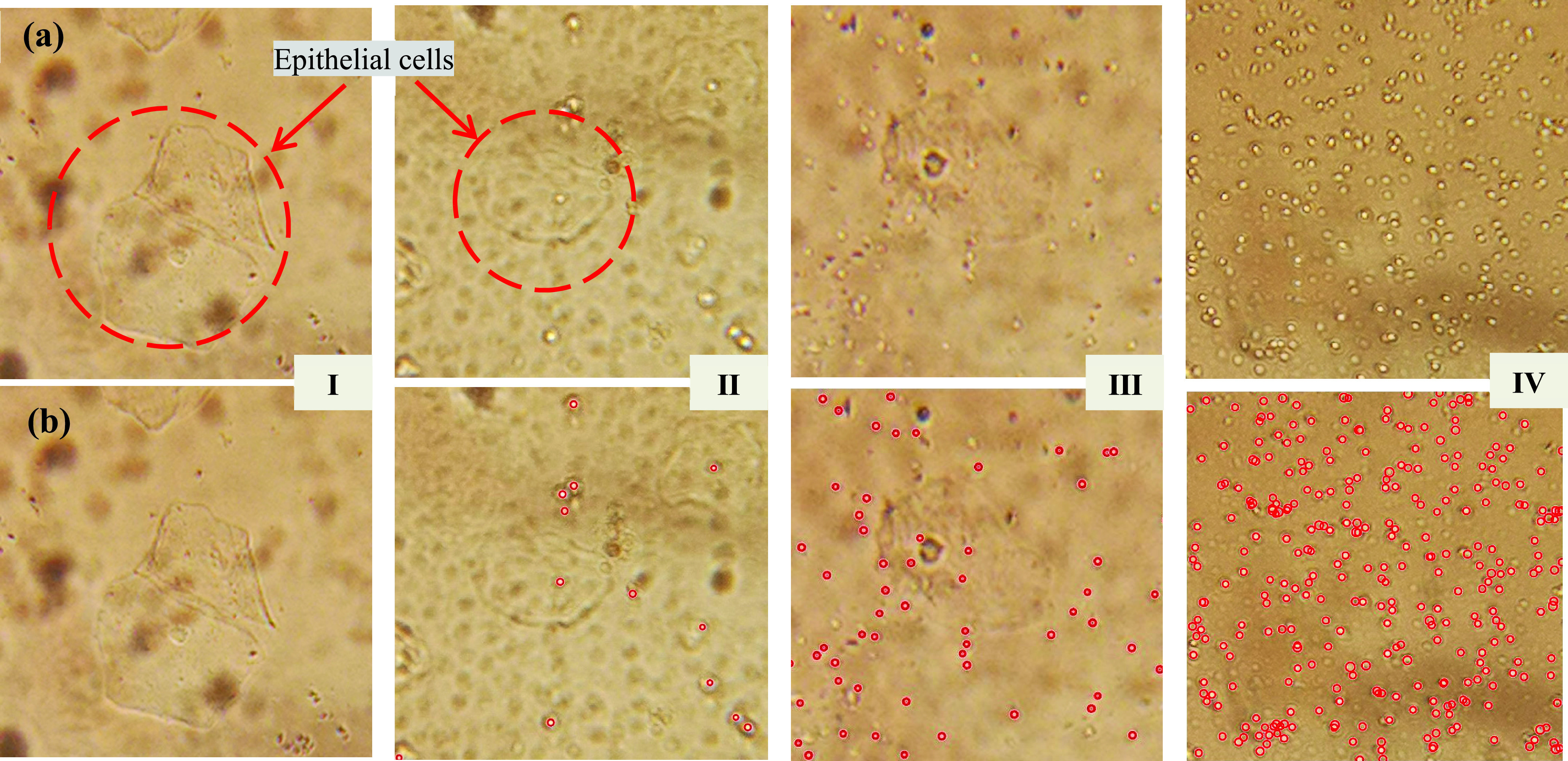
Clinical samples. (a) Original images of four different levels of clinical samples. (b) Images after recognition and counting.

Simultaneously, four clinical samples from Jiangbin hospital (Zhenjiang, China) were tested by the proposed method, two of which were III and IV. Repeated experiments were performed on the four samples, and the results showed that the four diagnoses were consistent, as shown in Table 2. At the same time, figure 8 (a) indicates the difference between the four grades of clinical samples. There are almost no cocci in normal leucorrhea, only a large number of epithelial cells and bacteria, while there are a large number of cocci and other miscellaneous bacteria in abnormal leucorrhea. Because there is a significant difference between the size of cocci and epithelial cells, and the cocci have prominent borders and high-brightness centers, it is easy to identify them. Figure 8 (b) is the image after identifying the cocci.

**TABLE 2:** Results of Clinical Samples Diagnosis

Normal sample 1			Normal sample 1		Patient sample 1		Patient sample 2	
Age	37		26		48		45	
	Number of Coccus/HFP	Mobile diagnosis	Number of Coccus/HFP	Mobile diagnosis	Number of Coccus/HFP	Mobile diagnosis	Number of Coccus/HFP	Mobile diagnosis
Image 1	1	I	8	II	23	III	188	IV
Image 2	0	I	12	II	25	III	194	IV
Image 3	0	I	11	II	19	III	193	IV
Image 4	1	I	10	II	27	III	189	IV

Although this system has a certain recognition error, the error is only reflected in high concentrations, and it is usually difficult for human samples to reach high concentrations. Therefore, the diagnosis of vaginal inflammation is feasible.

## IV. Conclusion

In this paper, we designed a hand-held zoom micro-imaging system which is based on a microfluidic chip. The combination of the device and smartphone can clearly see the morphological differences between cocci and bacillus, and its resolution can be less than 3 microns. At the same time, the recognition algorithm of APP we designed can also accurately determine the inflammation level, with an accuracy rate of 95%. In addition, the clinical samples were tested by the proposed detection system, and the results were consistent with the clinical diagnosis. In general, it is feasible to use this method to detect the health of women’s vaginal environment and can achieve the purpose of rapid detection at home. Still, for the optimization of algorithms, we need to continue to study in the future.
